# Development of soil-less substrates capable of degrading organic nitrogen into nitrate as in natural soils

**DOI:** 10.1038/s41598-022-04873-0

**Published:** 2022-01-17

**Authors:** Jamjan Meeboon, Ryoya Nishida, Takashi Iwai, Kazuki Fujiwara, Masao Takano, Makoto Shinohara

**Affiliations:** 1 Institute of Vegetable and Floriculture Science, National Agriculture and Food Research Organization (NARO), 360 Ano, Tsu, Mie 514-2392 Japan; 2 Graduate School of Environmental Studies, Nagoya University, Furo-cho, Chikusa-ku, Nagoya, Aichi 464-8601 Japan; 3 Kyushu Okinawa Agricultural Research Center, NARO, 2421 Suya, Koshi, Kumamoto 861-1192 Japan

**Keywords:** Biotechnology, Microbiology

## Abstract

Soil-less substrates are unable to catalyse nitrification because the addition of a high concentration of organic substances suppresses nitrification. We used a previously developed multiple parallel mineralization method, which enables the use of organic fertilizers in hydroponics, to support nitrification in soil-less substrates. In this method, microorganisms immobilized on porous substrates produced inorganic nitrate from organic substances, as in a natural soil. Phosphate and potassium ions were also released. Microorganisms produced nitrate from organic substances when immobilized on polyurethane resin, rockwool, vermiculite, oyster shell lime, and rice husk charcoal. The optimal amount of organic substance added daily to 100 mL of substrate held 6 mg of organic N. The optimal incubation temperature ranged from 25 to 42 °C. A high relative humidity (51% or higher) was more suitable than drier conditions (20%). The optimal amount of fish fertilizer added to the substrate was 6 mg organic N. The lower the C/N ratio of the organic substance, the better the result. Vegetable plants grew well on inoculated substrates but not on uninoculated substrates. These results show that soil-less substrates can be used to create artificial soils in which plants can be grown with the addition of organic fertilizer, as in a natural soil.

## Introduction

Soil is used to produce 98.8% of the calories in the world’s food^1^. It is common to use organic fertilizers made from food residues and composted livestock manure. In soil, a two-step degradation process (ammonification followed by nitrification) operates to make nitrogen available to plants^2,3^. Many crop plants require this nitrate–N, and fail to grow if nitrate is not supplied^4,5^. However, nitrification is difficult to achieve in water or soil-less substrates^6–11^, which have an impoverished microbial community compared with soil, and nitrification tends to be inhibited in the presence of high concentrations of organic substances such as l-threonine, l-valine, and fish-based soluble fertilizer^12–14^. If organic fertilizer is mixed into a soil-less substrate, such as regolith with a poorly developed microbial community, nitrate is not generated^6^.

Shinohara et al. developed a hydroponic technology that can degrade organic fertilizers into inorganic nutrients in water^14^. This “multiple parallel mineralization” (MPM) method allows co-culturing of nitrifying bacteria with heterotrophic microorganisms in water even in the presence of organic substances. Inoculation of microorganisms in an MPM system may allow both ammonification and nitrification to proceed, even in soil-less substrates. Unlike water, soil-less substrates have an aerobic particle surface, which can support the development of communities of nitrifying bacteria, which are generally aerobic^15^. If the soil-less substrate contains various minerals, it may have a positive effect on microbial activity.

Here, we created soil-like media from soil-less substrate by using the MPM technique to immobilize microorganisms on a non-soil carrier, where they were able to degrade organic N into nitrate–N, and tested plant growth in these media. To the best of our knowledge, this is the first use of the MPM method in a hydroponic system with soil-less substrates. We hypothesized that an optimal combination of substrate, organic fertilizer, temperature, and relative humidity (RH) during incubation would produce a substrate capable of supporting normal plant growth.

## Methods

The study was divided into three phases (Table S1). First, we examined potential carriers for the microbial communities and selected the most suitable carrier for subsequent experiments. Second, we tested a variety of conditions (temperature, amount of organic material, and average RH during incubation) to determine the optimal conditions for nitrification. Third, we determined the microbial taxa that were present in the medium and the effects of the optimal conditions on the growth of several crop plants.

### Selection of carriers

We tested 12 soil-less substrates: rockwool (grain size 3 to 7 mm; Grodan, Roermond, Netherlands; bulk density [ρ] 219.7 g/L); vermiculite (Nitto Hiruishi, Ianabe, Japan; ρ 147.6 g/L); pumice (Setogahara Kaen, Midori, Japan; ρ 408.7 g/L); perlite (Katanagawa Heiwa Nouen, Kanuma, Japan; ρ 60.0 g/L); coconut husk (Yoshimoto Nousan, Nangoku, Japan; ρ 78.0 g/L); 5 polyurethane resins (7.5 mm, Inoac Corporation, Nagoya, Japan: AQ-20, ρ 22.7 g/L; AQ-15, ρ 18.5 g/L; AQ-14, ρ 21.3 g/L; Mixel GR, ρ 41.0 g/L; Mixel GP, ρ 32.0 g/L); oyster shell lime (OSL; Suzuki Yuukan Center, Miyagi, Japan; ρ 693.8 g/L); and rice husk charcoal (Komeri, Niigata, Japan; ρ 110.2 g/L). Table S2 summarizes the physical characteristics of the substrates. We packed 100 mL of each material into an open-bottom tube made from the inverted top half of a 350-mL plastic soft-drink bottle (EBM, Tsubame, Japan), added 1 g of powdered bark compost (Sanyo bark, Sanyo Chip Kogyo, Shimonoseki, Japan) as the microbial inoculum, and then rinsed the materials with 100 mL of distilled water. We added 1 mL of 10% w/v fish fertilizer diluted with distilled water, and then incubated the tubes for 24 h in the dark at 25 °C in an incubator (CN-25C; Mitsubishi Electric, Tokyo, Japan). The next day, the carriers were rinsed with 100 mL of distilled water and the concentrations of ammonium, nitrite, and nitrate in the leachate were measured. The operation of adding fish fertilizer, incubating overnight at 25 °C, and rinsing with water was repeated daily for 2 weeks. Three tubes were used per experiment.

### Determination of optimal conditions

To immobilize microorganisms on the carriers^16^, we packed a weight equivalent to 100 mL of rockwool carrier (22 g; grain size 3 to7 mm; Grodan, ρ 219.7 g/L) in a tube and added 1 g of powdered bark compost (Sanyo bark), as inoculum (Fig. S1). We then rinsed the carriers with 100 mL of distilled water, added 1 mL of 10% w/v fish-based soluble fertilizer (Yaizu Suisankagaku Industry, Yaizu, Japan) diluted with distilled water, and incubated the tubes for 24 h in the dark at 25 °C in the CN-25C incubator. The next day, the carriers were rinsed with 100 mL of distilled water and the concentrations of ammonium, nitrite, and nitrate in the leachate were measured. The operation of adding fish fertilizer, incubating overnight at 25 °C, and rinsing with water was repeated daily for 2 to 3 weeks until nitrate was detected in the leachate. After a microbial community was established by completing this operation, the rockwool was used as a microorganism carrier in subsequent experiments. The chemical properties of the leachates from the carrier were measured daily for 30 days. Rockwool that was not inoculated with microorganisms but that was otherwise treated in the same manner was used as the control in subsequent experiments. These experiments were each performed in three tubes.

The following experiments were performed using tubes of rockwool carrier in which the addition of 6 mg N of fish fertilizer, overnight incubation at 25 °C, and rinsing with 100 mL of distilled water were repeated daily for 2 weeks until nitrate was detected in the leachate. After the detection of nitrate, we repeated the addition of fish fertilizer, overnight incubation, and rinsing with water daily for an additional 2 weeks in each experiment. All experiments were each performed in three tubes. To investigate the effect of excess organic substances on the production of inorganic N, we added 1 to 10 mL of 10% w/v fish fertilizer diluted with distilled water per tube daily so as to add 6 to 60 mg organic N. After incubation overnight, each tube was rinsed with 100 mL of distilled water daily.

In the subsequent experiments, we added 6 mg N of fish fertilizer (which provided the optimal results in the previous stage of the experiment). To determine the optimal incubation temperature, tubes were incubated at 15, 20, 25, 30, 37, 42, or 45 °C. To determine the optimal RH, tubes with materials that exhibited nitrification were incubated in incubators at an RH of 20% (WFO-600ND, Eyela, Tokyo, Japan), 51% (MLR-352, Panasonic, Kadoma, Japan), or 92% (CN-25C) at 25 °C. The amounts of inorganic N, ammonium, nitrite, and nitrate in the leachates were measured under each experimental condition.

The following experiments were performed using tubes of rockwool carrier in which the addition of fish fertilizer, overnight incubation, and rinsing with water had been repeated daily for 2 to 3 weeks until nitrate was detected in the leachate. To compare the effects of different organic substances, we added fish fertilizer (6% N w/w, C/N ratio 2.9), corn steep liquor (CSL, OAT Agrio, Tokyo, Japan; 3.3% N w/w, C/N ratio 4.8), or rapeseed oil cake (Sun and Hope, Kitakyushu, Japan; 6% N w/w, C/N ratio 6.9) as sources of organic N in amounts equivalent to 6 mg N per tube and then incubated the tubes overnight in the dark at 25 °C. The tubes were then rinsed with 100 mL of distilled water, followed by the addition of organic substances and incubation, daily for 3 weeks. The amounts of inorganic N, ammonium, nitrite, and nitrate in the leachates were then measured. These experiments were each performed in three tubes.

### Identification of microbes and confirmation of plant growth

To estimate the microbial density, we added 1 g (fresh weight) of the sample carrier (rockwool) to 9 mL of sterilized water and vortexed the mixture for 5 min; we then applied 100 μL of the sample to 1/10 NA medium (0.8 g/L Difco nutrient broth [Becton Dickinson, Franklin Lakes, NJ, USA] + 15 g/L agar [Fujifilm Wako, Osaka, Japan]) using the dilution plate technique. We incubated the plates at 25 °C for 1 week in the dark and counted the colonies. To detect and count the nitrifying bacteria, we used two antibody-based determination kits (Kenshutsu-kun and Spira-kun; Yakult, Tokyo, Japan).

We analysed the microbial phase in the rockwool carrier^17^. We prepared tubes filled with 100 mL of rockwool by rinsing the material with 100 mL of distilled water; we added 1 g of bark compost on the top as inoculum and 1 mL of 10% w/v fish fertilizer diluted with distilled water as the organic substance, and then incubated the tubes in the dark overnight at 25 °C. The next day, the tubes were rinsed with 100 mL of distilled water and then 1 mL of 10% w/v fish fertilizer was again added. The addition of fish fertilizer, incubation overnight, and rinsing with water was repeated daily for 3 weeks until nitrate was detected in the leachate. Granules of rockwool that carried a microbial community were sent to TechnoSuruga Laboratory Co., Ltd. (Shizuoka, Japan) for amplicon sequence analysis^17^. Total DNA was extracted using the ISOIL for Beads Beating kit (Nippon Gene Co., Ltd., Toyama, Japan). Total DNA was purified with the DNeasy PowerClean Pro Cleanup Kit (Qiagen, Hulsterweg, The Netherlands). The V3–V4 regions of bacterial and archaeal 16S rRNA were amplified using the Pro 341F/805R primers and dual-index method^18,19^.

### Identification from sequences

Sequence reads were analysed manually using the Ribosomal Database Project (RDP) Multiclassifier tool, which is available from the RDP website (http://rdp.cme.msu.edu/classifier/)^20^. Bacterial species were identified from sequences in Metagenome@KIN v. 2.2.1 analysis software (World Fusion, Japan) and in the TechnoSuruga Lab Microbial Identification database DB-BA 13.0 (TechnoSuruga Laboratory, Shizuoka, Japan) with homology for ≥ 97%^21^. We also analysed the microbial phase of the bark compost and the MPM liquid culture solution to provide a comparison. To prepare the MPM liquid culture solution, we placed 100 mL of sterilized distilled water with 1 g of bark compost as inoculum and added fish fertilizer to a final concentration of 1 g/L in a flask and cultured the solution on an orbital shaker (120 rpm) for 2 weeks at 25 °C in the dark. The culture solution was then centrifuged at 10,000×*g* for 5 min. The cell pellet was analysed by TechnoSuruga as described earlier in this section.

On rockwool that carried a microbial community, we sowed 5 seeds of komatsuna (*Brassica rapa* var. *perviridis*, ATU121 ‘Misaki’; Sakata Seed Corporation, Yokohama, Kanagawa, Japan), then added 0.1 g of fish fertilizer to each carrier and incubated the carriers in an MLR-352 chamber at 25 °C under a 12-h light/dark cycle for 24 h, and then rinsed the carrier with 100 mL of distilled water. We repeated the addition of fish fertilizer, incubation, and rinsing daily for 11 days. We also grew saladana lettuce (*Lactuca sativa* var. *capitata* ‘Santa Clara’; Tohoku Seed Co. Ltd., Utsunomiya, Tochigi, Japan), radish (*Raphanus sativus* var. *sativus*, ‘Akamaru Hatsuka Daikon’; Nihon Nousan Shubyo Co. Ltd., Kamiina, Nagano, Japan), turnip (*Brassica rapa* var. *glabra*, ‘Shogoin Kabu’; Atariya Nouen, Katori, Chiba, Japan), and ball lettuce (*Lactuca sativa* ‘Melbourne-MT’; Tohoku Seed Co. Ltd.) on rockwool and nursery soil (Naeichiban; Sumirin Agro-Products, Aichi, Japan) in a greenhouse of the Institute of Vegetable and Floriculture Science in Tsu, Mie Prefecture, from September to December 2013. During this period, photoperiod ranged from 305.9 to 371.5 h/month, with daily average outside temperatures of 6.0 to 23.5 °C, daily minimum outside temperatures of 1.7 to 19.4 °C, and daily maximum outside temperatures of 10.9 to 28.3 °C. Light and temperature were not controlled. Three pots per treatment were examined. We conducted these experiments in accordance with relevant institutional, national, and international guidelines and legislation. All plant seeds used were commercially available.

### Methods for leachate analysis

We used Reflectoquant tests for the ammonium (Merck, Darmstadt, Germany; catalogue number 116892), nitrite (116973), nitrate (116971), phosphate (116978) and potassium (117945) measurements^14^. We added and mixed the supplied reagents according to the manufacturer’s instructions for each kit, submerged the test strip, and measured the degree of colour development using a reflective photometer (RQflex plus; Merck)^14^. When the leachate contained nitrite, we added 150 μL of 10% (w/v) amidosulfuric acid (Kanto Chemical, Tokyo, Japan) to 5 mL of the leachate before measuring nitrate, and mixed the solution to eliminate the influence of nitrite. pH and electrical conductivity (EC) were measured using a pH meter (C-73, AZ-ONE, Osaka, Japan) and an EC meter (Twin Cond, Horiba, Kyoto, Japan), respectively.

## Results

### Selection of carrier

**Figure 1:**
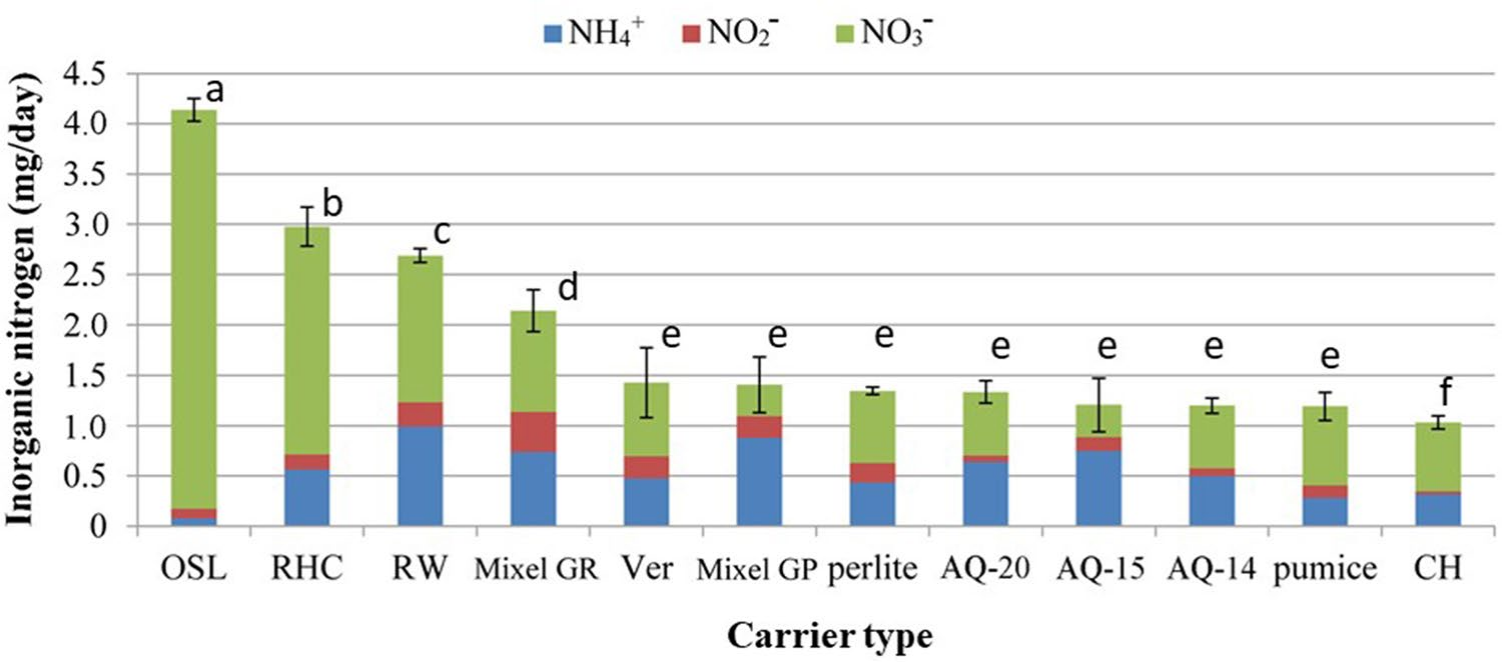
Nitrogen mineralization by the microbial communities on different carriers. Carriers: *OSL* oyster shell lime, *RHC* rice husk charcoal, *RW* rockwool, *Mixel GR* polyurethane Mixel GR, *Ver* vermiculite, *Mixel GP* polyurethane Mixel GP, *AQ-20* polyurethane AQ-20, *AQ-15* polyurethane AQ-15, *AQ-14* polyurethane AQ-14, *CH* coconut husk. Values are means ± SD (*n* = 3). Bars labelled with different letters differ significantly (Fisher’s protected LSD test, *P* < 0.05).

Nitrate–N was produced from all 12 porous carriers that carried a microbial community (Fig. 1). OSL, rice husk charcoal, rockwool, and Mixel GR produced significantly higher total inorganic N than the other carriers, including large amounts of nitrate (53% to 96% of the total inorganic N). OSL achieved a net N mineralization of 69%, but phosphate could not be detected (data not shown). No physical properties seemed to be significantly correlated with nitrate production efficiency (Fig. S2). Since OSL and rice husk charcoal are natural products and it is therefore difficult to obtain products of constant quality, we conducted our subsequent experiments using rockwool.

### Determination of optimal conditions

**Figure 2:**
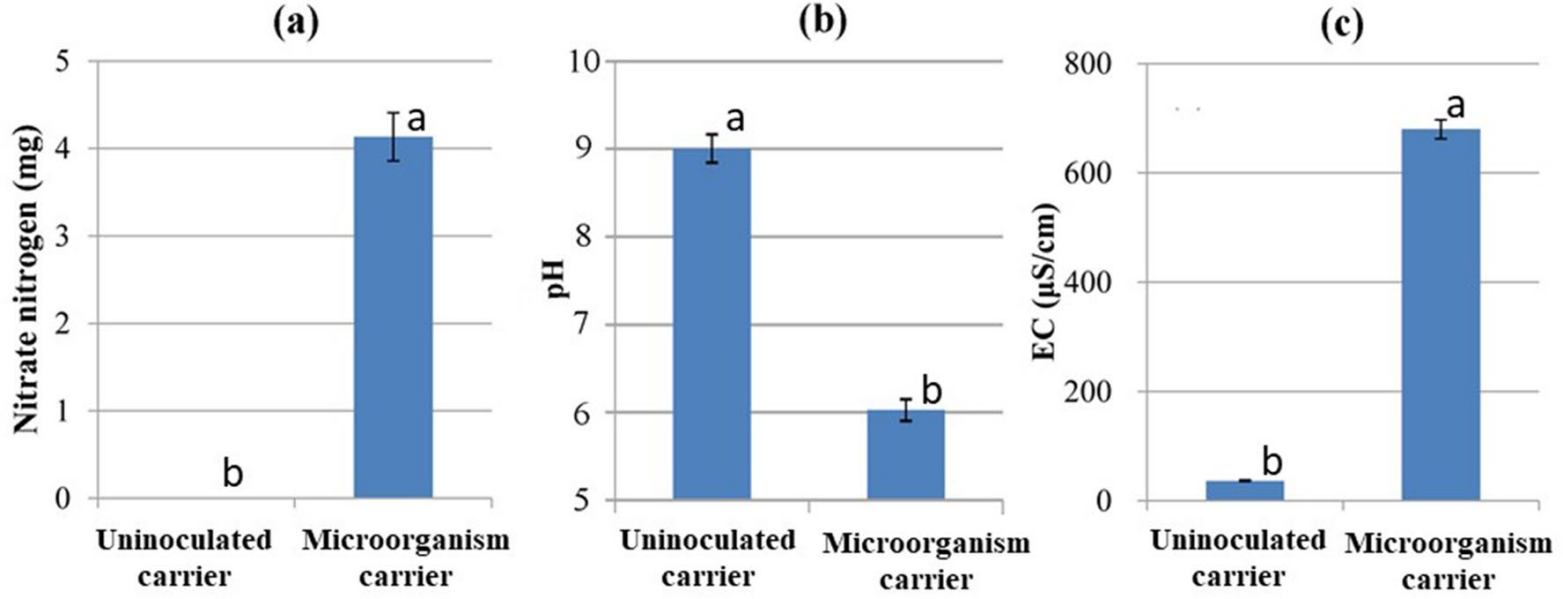
Comparisons between leachates from the inoculated rockwool carrier and leachates from the uninoculated rockwool. Comparisons of (**a**) nitrate–N, (**b**) pH, and (**c**) electrical conductivity (EC) of the leachate solutions from 100 mL of inoculated or uninoculated rockwool. The carrier received 1 g of bark compost as inoculum. All tubes received 0.1 g of fish-based soluble fertilizer every day and were rinsed with 100 mL of water the next day. Values are means ± SD (*n* = 3). Values of a property labelled with the same letter do not differ significantly (Fisher’s protected LSD test, *P* < 0.05).

The chemical properties of the leachates from the inoculated carrier differed significantly from those from uninoculated rockwool (Fig. 2). The generation of nitrate from the carrier remained high for 2 weeks after its start (Figs. S3, S4). The low pH of leachate from the carrier (which changed from slightly alkaline in the uninoculated carrier to slightly acidic) indicates that more nitrate–N was generated than ammonium-N (Figs. 2, S3). No nitrate or nitrite was detected in the leachate from the uninoculated rockwool; only ammonium-N and potassium were detected (data not shown). The higher EC of the carrier than of the uninoculated rockwool indicates that more inorganic ions were produced. By 32 days, 1.7 mg of potassium and 1.4 mg of phosphate were generated in the leachate from the carrier (Fig. S5). No phosphate was detected in the leachates from the uninoculated rockwool (data not shown).

**Figure 3:**
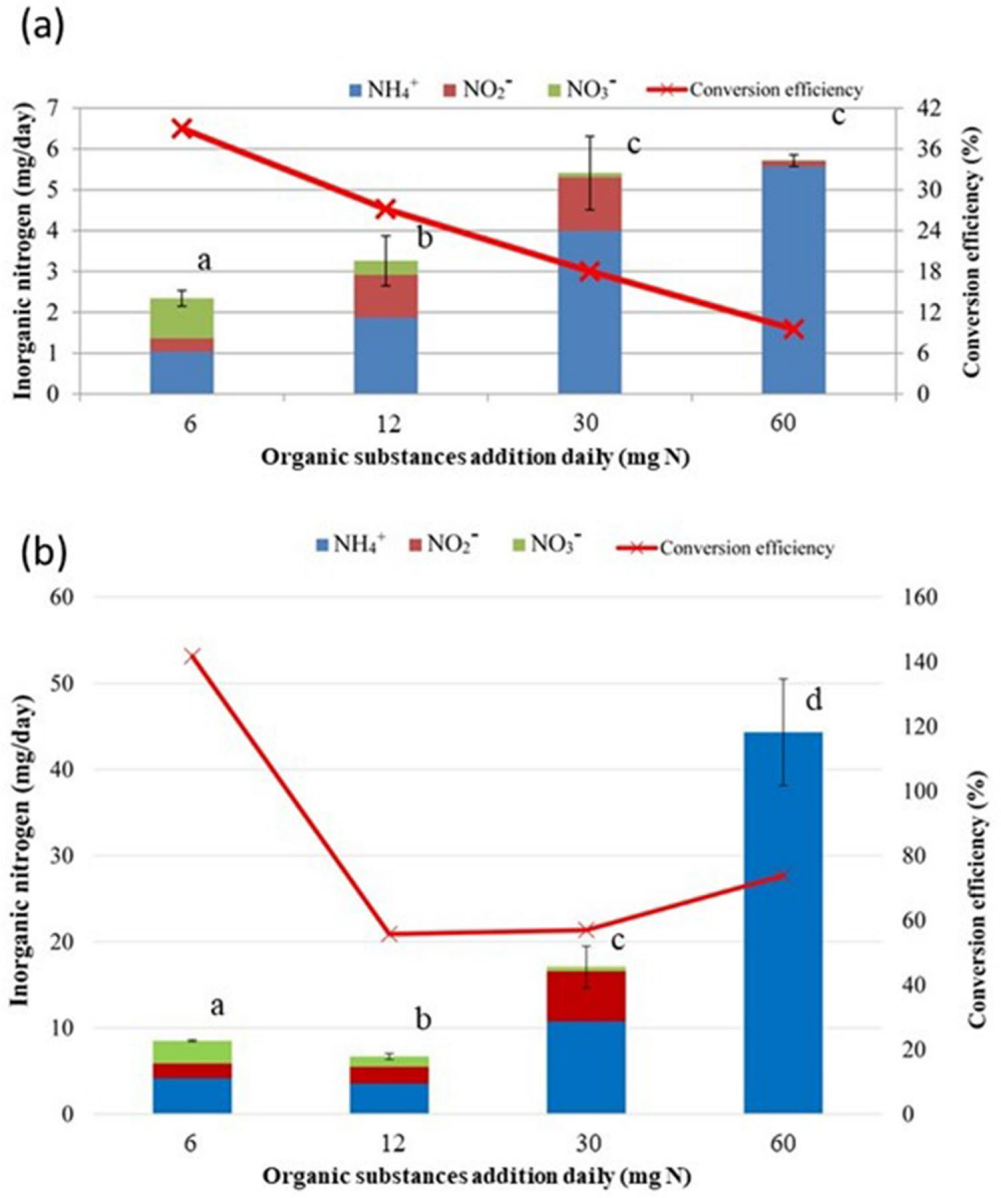
Effect of excessive addition of an organic substance and the resulting inorganic N composition. (**a**) We added 6 mg N of fish fertilizer daily for 2 weeks until nitrate was detected in the leachate and then added 6 to 60 mg N of fish fertilizer for 2 weeks. (**b**) Immediately after addition of bark compost, we added the indicated amounts of fish fertilizer for 2 weeks. The composition of inorganic N in the leachate was then measured. Values are means ± SD (*n* = 3). Bars labelled with the same letter do not differ significantly (Fisher’s protected LSD test, *P* < 0.05).

We investigated the effect of excessive addition of organic substances on the production of inorganic N. When we increased the amount of added organic N (as fish fertilizer), the amount of inorganic N in the leachate increased (Fig. 3a). However, the amount of nitrate–N and the efficiency of the conversion to inorganic N decreased. When 60 mg N of organic N was added per tube immediately after addition of the bark compost, no nitrate and almost no nitrite were detected (Fig. 3b).

**Figure 4:**
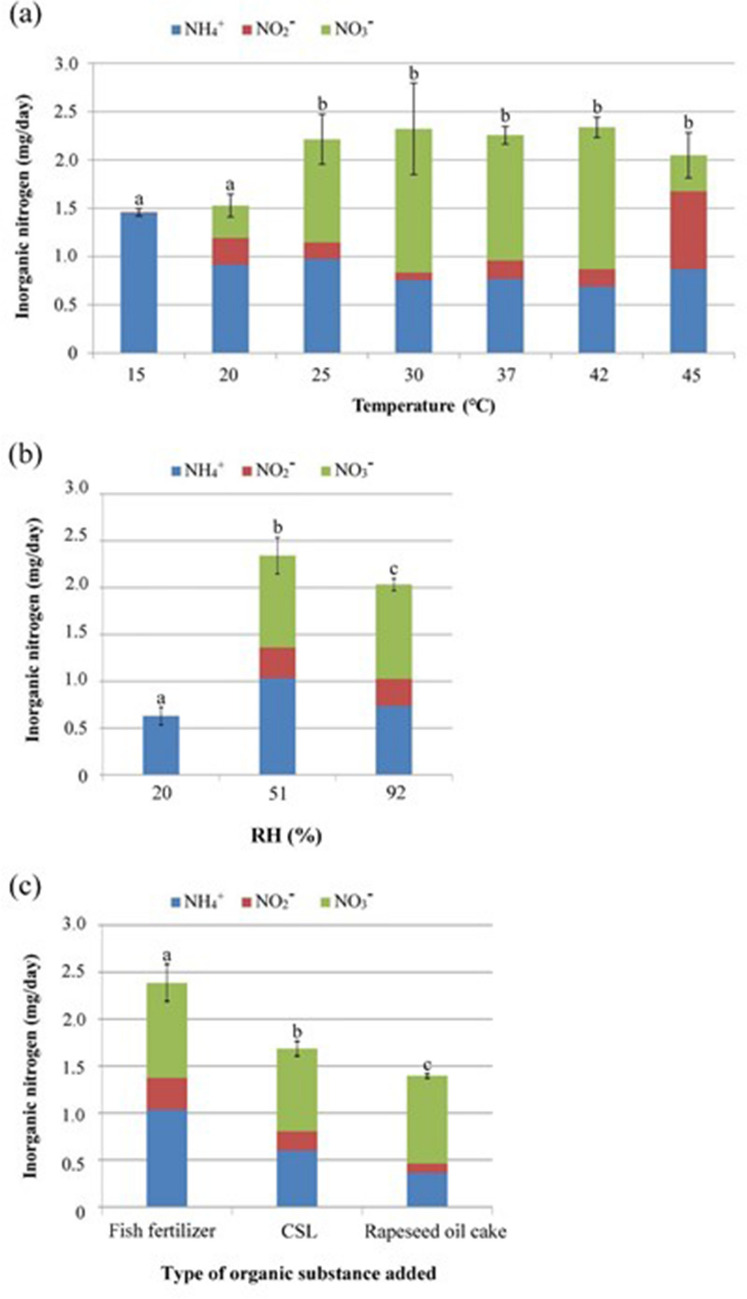
Determination of optimal conditions for N mineralization in the leachate from rockwool treated with organic substances. Bars labelled with the same letter do not differ significantly (Fisher’s protected LSD test, *P* < 0.05). (**a**) Effect of incubation temperature on N mineralization with fish fertilizer. We measured the composition of inorganic N in the leachate following incubation at the indicated temperatures. Values are means ± SD (*n* = 3). (**b**) Effect of relative humidity (RH) during incubation on N mineralization with fish fertilizer. We incubated the inoculated carriers at the indicated RH and measured the composition of inorganic N in the leachate. Values are means ± SD (*n* = 3). (**c**) Effect of organic substance type on N mineralization. Values are means ± SD (*n* = 3).

We also investigated the effect of incubation temperature on the mineralization of organic N. The production of inorganic N was significantly lower at 15 and 20 °C than at higher temperatures, and almost no nitrite or nitrate was produced at 15 °C (Fig. 4a). The total inorganic N production and nitrate production were significantly higher at incubation temperatures from 25 to 42 °C, with no significant difference among these temperatures.

The effect of RH on the mineralization of organic N was statistically significant (Fig. 4b). At 20% RH, ammonium was generated, but no nitrite or nitrate, and the inside of the carrier was moist, but the surface was dry. At 51% and 92% RH, the amounts of inorganic N and nitrate produced were significantly higher, and inorganic N reached its maximum at 51% RH. Fish fertilizer was mineralized significantly better than CSL and rapeseed oil cake (Fig. 4c).

### Identification of microbes and confirmation of plant growth

Changes in the density of microorganisms on the carrier were investigated in NA medium. The population density increased dramatically from about 2 × 10^6^ cfu/g (in 1/10 NA medium) before incubation to about 60 × 10^6^ cfu/g after 3 days of incubation (Fig. S6). Thereafter, the bacterial density stabilized. One day after the start of incubation, we detected nitrite and nitrate, which we hypothesize were present in the bark compost, but after 2 and 3 days only ammonia was produced. Nitrite began to appear at 8 days, and nitrate at 14 days. We attempted to detect and count nitrifying bacteria using two antibody-based determination kits, but the numbers of bacteria were below the detection limit (1.0 × 10^5^ cells/g; data not shown).

**Figure 5:**
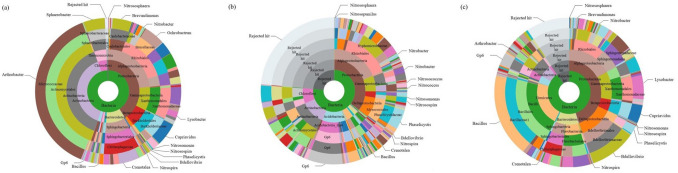
Microbial phase analysis of the inoculated carriers, uninoculated material, and liquid “multiple parallel mineralization” (MPM) culture. Sunburst display of species annotation. The innermost circles represent the highest level in the taxonomic hierarchy (i.e., kingdom); moving towards the outer circles represents a move towards lower taxonomic levels (with genus as the outermost level). Sector areas indicate the relative proportions of operational taxonomic unit annotation results. (**a**) Rockwool as the microorganism carrier (32,130 sequences). (**b**) Bark compost as the microbial inoculum (38,114 sequences). (**c**) Liquid culture MPM solution with 10 g/L of bark compost as the microbial inoculum and 1 g/L of fish fertilizer incubated in a flask for 2 weeks at 25 °C, with shaking at 120 rpm (50 381 sequences).

Analysis detected 32,130 distinct sequences in the carrier (DNA Database of Japan [DDBJ] accession number DRR316169), 38,114 in the bark compost (DRR316170), and 50,381 in the MPM culture solution (DRR316171) (Table S3). *Arthrobacter* (38.1%, 12,254 sequences), *Cupriavidus* (5.1%, 1628 sequences), *Ochrobactrum* (4.7%, 1499 sequences), *Sphaerobacter* (4.6%, 1476 sequences), *Crenotalea* (4.5%, 1461 sequences), and *Brevundimonas* (4.3%, 1381 sequences) were relatively common in the microbial phase of the carrier (Fig. 5a). *Arthrobacter* was the dominant taxon in the carrier (Fig. 5a); we found no *Arthrobacter* in the sequence data of the bark compost (data not shown; Fig. 5b), but detected 21 sequences (< 0.1%) in the MPM culture solution (data not shown; Fig. 5c). *Bacillus* (18.0%, 9074 sequences) was the dominant taxon in the MPM culture solution; in contrast, only 15 sequences (< 0.1%) were detected in the carrier and 121 (0.3%) in the bark compost. Nitrite-oxidizing *Nitrobacter* was detected in 102 sequences (0.3%) in the carrier, 196 (0.5%) in the bark compost, and 133 (0.3%) in the MPM culture solution. Nitrite-oxidizing *Nitrospira* was detected in 4 sequences (< 0.1%) from the carrier, 34 (< 0.1%) in bark compost, and 29 (< 0.1%) in MPM culture solution. Ammonium-oxidizing bacteria, such as *Nitrosomonas*, were detected in 77 sequences (0.2%) in the carrier, 2 (< 0.1%) in the bark compost, and 52 (0.1%) in the MPM culture solution.

**Figure 6:**
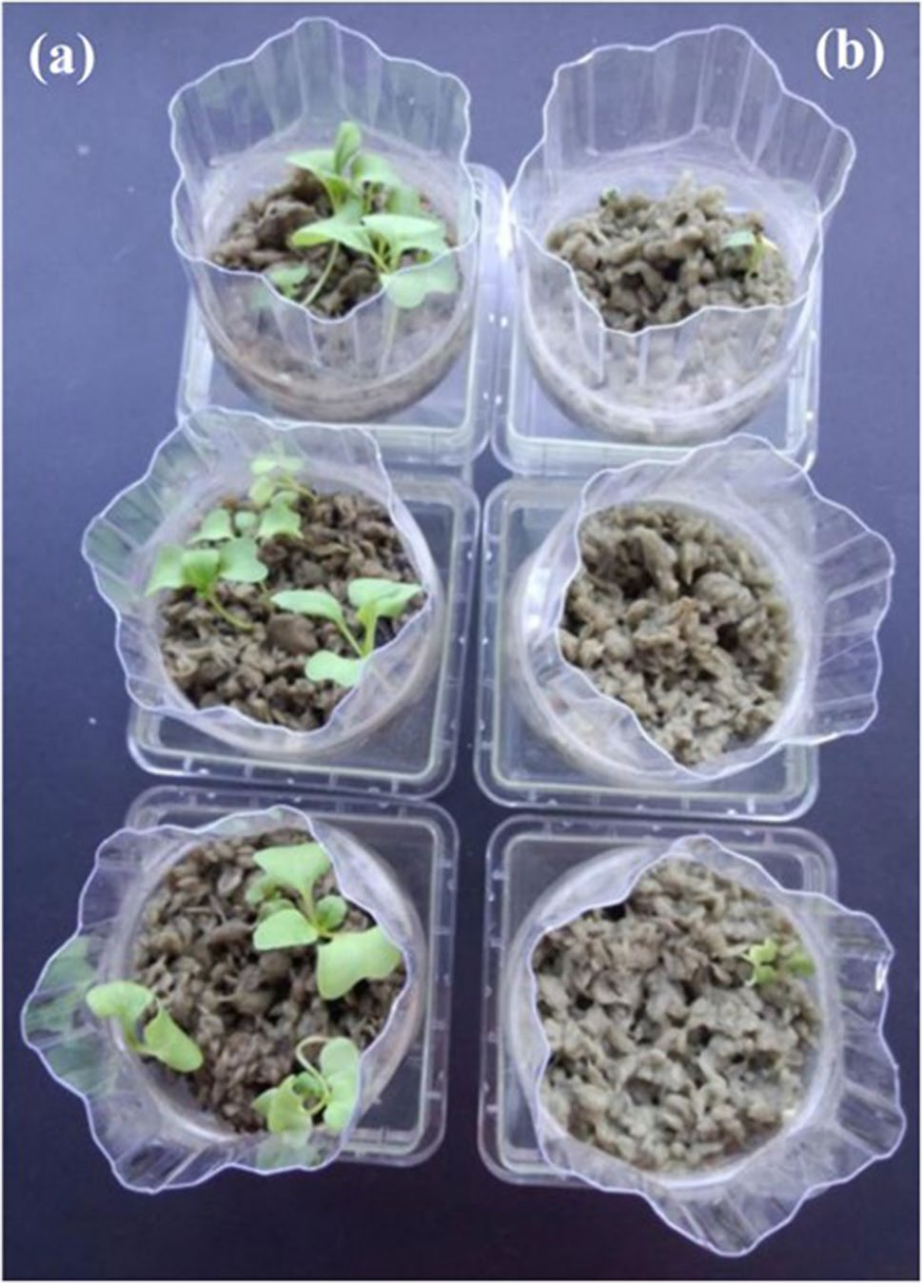
Plant growth on the inoculated carrier and the uninoculated materials. Komatsuna seeds were sown in (**a**) the inoculated carrier that had acquired the ability to degrade organic substances into inorganic nutrients or (**b**) uninoculated rockwool, and then grown at 25 °C. The photo shows plants at 11 days after sowing.

Komatsuna seedlings grew well in the inoculated rockwool but not in uninoculated rockwool (Fig. 6a). When saladana lettuce was grown with the addition of fish fertilizer, growth in the carrier was comparable to that in soil, whereas that in uninoculated rockwool was poor (Fig. S7a–c). In addition, radishes, turnips, and ball lettuce showed the same or similar growth as in soil (Fig. S7d–m).

## Discussion

Soil-less substrates usually do not allow organic substances to be used as fertilizer. In conventional hydroponics, organic fertilizers typically have phytotoxic effects that lead to poor plant growth and are difficult to use because of the lack of a microbial community that degrades and mineralizes organic N compounds into nitrate–N^6,7,14,22^. If we want to grow crops with organic fertilizer in soil-less substrates, we must therefore achieve the ability to degrade organic N into nitrate–N^6,14^. Here, we achieved nitrification in soil-less substrates by immobilization of microbial communities on a carrier (Fig. 2). We hypothesized that if a microbial ecosystem capable of nitrification could be constructed, the phytotoxic effects of organic fertilizers could be eliminated, and confirmed this hypothesis in several vegetable crops (Figs. 6, S7), but the mechanism is unknown. In future research, it will be necessary to investigate how the establishment of nitrification eliminates the phytotoxicity.

We tested carriers composed of artificial resins (polyurethanes), mineral-derived materials (vermiculite, perlite, and rockwool), biological-derived materials (OSL and charcoal), and natural materials (coconut husk). Even on these soil-less substrates, it proved possible to induce the same inorganic nutrient–producing ability that occurs in natural soil. This shows the possibility of creating artificial soils with a designed pore size, particle size, water retention, and ion-exchange capacity. We investigated the physics of the porous carrier and found no physical properties that would affect the efficiency of conversion to nitrate–N (Fig. S2). If properties that are strongly correlated with the efficiency of conversion can be found, it will help researchers to produce artificial soils that increase plant growth. If such properties could be identified in a natural soil, they might also be manipulated to increase crop productivity. It will be necessary to develop a physical evaluation method whose results are strongly correlated with efficiency of conversion to nitrate–N. OSL produced significantly more inorganic N than the other substrates (Fig. 1), but the leachate did not contain phosphate (data not shown). These results suggest that any inorganic phosphate produced in this system reacted with calcium carbonate, the main component of OSL, keeping phosphate out of the leachate^23^. Minerals in OSL and charcoal may improve microbial activity, resulting in higher production of inorganic N^24,25^. The cation exchange capacity of rockwool is almost nil^26^, so mineral supply from the rock wool appears to have little effect.

Nitrate production at high levels occurred consistently from day 13 onwards (Fig. S3). The nitrification ability of inoculated carriers was significantly higher between 25 and 45 °C than at lower temperatures (Fig. 4a). In natural soils, the conversion of organic N to inorganic N increases as temperatures increase from 5 to 35 °C^27–30^ and is highest at 35 °C^30^. In soil, the amount of inorganic N is influenced more by soil temperature than by soil moisture content^31^, but our results show that the N mineralization ability decreased significantly in dry air (Fig. 4b). Nitrate–N was produced at RH ≥ 51% but not at 20% RH (Fig. 4b). The inside of the carrier at 20% RH was moist but the surface was dry. We removed the top 1 cm of the rockwool carrier incubated at 51% RH and repeated the addition of fish fertilizer, followed by overnight incubation at 51% RH and daily rinsing with water, but did not detect nitrate (data not shown). This result suggests that most nitrifying bacteria inhabit the surface layer of the carrier and are inactive in the moist but anaerobic inner layer. In future research, it will be necessary to separately sample the surface and lower layers of the carrier to test this hypothesis. The N mineralization activity was significantly higher at 51% RH than that at 92% RH. Excessive humidity may have led to wet anaerobic conditions in the porous carrier, reducing the activity of the nitrifying bacteria^32^. The 1/10 NA medium that we used to determine bacterial density (Fig. S6) is frequently used to culture common soil microorganisms^33^, but this assay probably excluded nitrifying bacteria, whose growth is significantly inhibited on media that contain organic substances^34^. We detected nitrate and nitrite, which we hypothesize were derived from the bark compost, on day 1, but not on days 2 or 3 (Figs. S3, S4, S6). These results suggest that the nitrifying bacteria are present in the bark compost but that nitrification is temporarily suppressed by the addition of fish fertilizer^14^. The antibody-based determination kits that we used could not detect nitrifying bacteria. As nitrifying bacteria appear to be localized on the surface of the carrier and their densities are low, it is difficult to investigate how their density changes in the carrier.

When fish fertilizer with ≥ 12 mg N was added, the amount of inorganic N produced increased but the amount of nitrate–N produced decreased relative to that when we applied 6 mg N (Fig. 3a). These results suggest that the addition of excessive amounts of organic matter suppresses nitrification^14^.

The addition of fish fertilizer (C/N = 2.9) produced significantly more inorganic N than that of CSL (4.8) or rapeseed oil cake (6.9) (Fig. 4c). These results suggest that the higher the C/N ratio of the organic fertilizer, the lower the efficiency of inorganic N production. However, this hypothesis should be tested to ensure that the ratio, and not other factors such as the chemical composition of the organic matter, is responsible for this difference.

From the results of our comparison of temperature, RH, and the type and amount of organic matter, the optimal incubation temperature appears to range from 25 to 42 °C. The optimal amount of fish fertilizer added to the substrate was 6 mg organic N. The rate of recovery of inorganic N was higher from organic substances with a smaller C/N ratio.

A microorganism carrier could be used to produce inorganic fertilizers from organic substances. Adding an organic substance such as fish fertilizer to an inoculated carrier and rinsing it with water the next day could allow the inorganic nutrient solution to be collected daily^16^. Most inorganic fertilizers are chemically synthesized from minerals or the air using methods that require large amounts of energy. Our technique offers a new method for producing these nutrients from organic resources with little energy input. The optimal conditions here will be useful for guiding the development of such new methods.

We used bark compost as the inoculum, but bark composts are often aged on concrete and may therefore be free of nitrifying bacteria (data not shown). We confirmed that the bark compost we used was aged on soil. Readers who want to replicate or build on our study should therefore use organic farm soils that are definitely inhabited by nitrifying bacteria.

The fish fertilizer was used without sterilization. The bacterial densities of the fish fertilizer and rockwool determined by the dilution plate method using 1/10 NA were both very low, at 6.7 × 10^2^ cfu/g (data not shown). The results with the uninoculated carrier in Fig. 2 suggest that neither contains ammonia- or nitrite-oxidizing bacteria.

The microbial phase of the microorganism carrier was very different from those of the bark compost (microbial source) and the MPM liquid culture solution (Fig. 5). In place of the bark compost, which contains a multitude of microorganisms, we will try in future research to create a microorganism carrier with a small number of microbial species of those major genera and nitrifying bacteria. Artificial soil created with only a limited number of microorganisms may serve as, for example, a model for analysing plant–microbe interactions. The microbial phase of the microorganism carrier may change with temperature and RH and with the type and amount of organic substance added, so obtaining such data will contribute to the creation of future artificial soils.

Both plants such as komatsuna that prefer nitrate–N and plants such as lettuce that prefer ammonium–N^18^ grew well in the inoculated rockwool but not in uninoculated rockwool (Figs. 6, S7). These results suggest that nitrate production is important for the growth of both types of plant. Our experiments with radishes, turnips, and ball lettuce (Fig. S7) suggest that rockwool inoculated with MPM microorganisms can degrade organic substances such as fish fertilizer into inorganic nutrients, as in natural soil.

In summary, we demonstrated the ability of inoculated carriers to mineralize organic N into nitrate–N and their potential to replace natural soils. Technology to create artificial soils will help clarify the ideal physical and chemical properties of soils and contribute to increased food production. The good plant growth on the optimal substrate confirmed our hypothesis that the MPM method could be applied to solid substrates.

## Supplementary Information

Supplementary Information.

## Data Availability

Supplementary information for this paper is available in the DNA Database of Japan (DDBJ) using Accession Numbers DRA012652, DRR316169, DRR316170, and DRR316171.

## References

[1] FAO. *2018 FAO Statistical Databases*. Food and Agriculture Organization of the United Nations. http://apps.fao.org/ (2018).

[2] Dancer WS, Peterson LA, Chesters G (1973). Ammonification and nitrification of N as influenced by soil pH and previous N treatments. Soil Sci. Soc. Am. J..

[3] Alsanius BW, Wohanka W (2003). Root Microbial immobilization of ammonium and nitrate in relation to ammonification and nitrification rates in organic and conventional cropping system. Soil Biol. Biochem..

[4] Puritch GS, Barker AV (1967). Structure and function of tomato leaf chloroplasts during ammonium toxicity. Plant Physiol..

[5] Magalhaes JR, Huber DM (1991). Response of ammonium assimilation enzymes to nitrogen form treatments in different plant species. J. Plant Nutr..

[6] Miyata H, Ikeda H (1997). The Analysis Methods of Soil Environment.

[7] Atkin K, Nichols MA (2004). Organic hydroponics. Acta Hort..

[8] Stutte GW (1996). Nitrogen dynamics in the CELSS breadboard facility at Kennedy Space Center. Life Support Biosphere. Sci..

[9] Schwartzkopf SH, Stroup TL, Williams DW (1993). Anaerobically-processed waste as a nutrient source for higher plants in a controlled ecological life support system. SAE Trans..

[10] Mackowiak CL, Garland JL, Strayer RF, Finger BW, Wheeler RM (1996). Comparison of aerobically-treated and untreated crop residue as a source of recycled nutrients in a recirculating hydroponic system. Adv. Space Res..

[11] Garland JL, Mackowiak CL, Strayer RF, Finger BW (1997). Integration of waste processing and biomass production systems as part of the KSC Breadboard project. Adv. Space Res..

[12] Hockenbury MR, Grady CPL (1977). Inhibition of nitrification-effects of selected organic compounds. J. WPCF.

[13] Strauss EA, Lamberti GA (2000). Regulation of nitrification in aquatic sediments by organic carbon. Limnol. Oceanogr..

[14] Shinohara M (2011). Microbial mineralization of organic nitrogen into nitrate to allow the use of organic fertilizer in hydroponics. Soil Sci. Plant Nutr..

[15] Quoc BN, Wei S, Armenta M, Bucher R, Sukapanpotharam P, Stahl DA, Stensel HD, Winklera MKH (2021). Aerobic granular sludge: Impact of size distribution on nitrification capacity. Water Res..

[16] Shinohara, M. *Method for Producing the Carrier, Catalyst Column, and Solid Medium for Plant Growth Immobilized Parallel Mineralizing Microorganisms*. Japanese Patent Application Laid-Open No. 2010–88358 (in Japanese) (2010).

[17] Ohara T (2019). Identification of the microbial diversity after fecal microbiota transplantation therapy for chronic intractable constipation using 16s rRNA amplicon sequencing. PLoS ONE.

[18] Takahashi S, Tomita J, Nishioka K, Hisada T, Nishijima M (2014). Development of a prokaryotic universal primer for simultaneous analysis of Bacteria and Archaea using next-generation sequencing. PLoS ONE.

[19] Hisada T, Endoh K, Kuriki K (2015). Inter-and intra-individual variations in seasonal and daily stabilities of the human gut microbiota. Arch. Microbiol..

[20] Wang Q, Garrity GM, Tiedje JM, Cole JR (2007). Naive bayesian classifier for rapid assignment of rRNA sequences into the new bacterial taxonomy. Appl. Environ. Microbiol..

[21] Kasai C, Sugimoto K, Moritani I, Tanaka J, Oya Y, Inoue H, Tameda M, Shiraki K, Ito M, Takei Y, Takase K (2015). Comparison of the gut microbiota composition between obese and non-obese individuals in a Japanese population, as analyzed by terminal restriction fragment length polymorphism and next-generation sequencing. BMC Gastroenterol..

[22] Ikeda H, Osawa T (1981). Nitrate-and ammonium-N absorption by vegetables from nutrient solution containing ammonium nitrate and the resultant change of solution pH. J. Jpn. Soc. Hort. Sci..

[23] Hamester MRR, Balzer PS, Becker D (2012). Characterization of calcium carbonate obtained from oyster and mussel shells and incorporation in polypropylene. Mater. Res..

[24] Lee YH, Islam SMA, Hong SJ, Cho KM, Math RK, Heo JY, Kim H, Yun HD (2010). Composted oyster shell as lime fertilizer is more effective than fresh oyster shell. Biosci. Biotechnol. Biochem..

[25] Hamzah A, Hapsari RI, Priyadarshini R (2017). The influence of rice husk and tobacco waste biochars on soil quality. JDMLM.

[26] Lemaire F (1995). Physical, chemical and biological properties of growing medium. ISHS Acta Hort..

[27] Stanford G, Frere MH, Schwaninger DH (1973). Temperature coefficient of soil nitrogen mineralization. Soil Sci..

[28] Addiscott TM (1983). Kinetics and temperature relationship of mineralization and nitrification in Rothamstead soils with differing histories. J. Soil Sci..

[29] Ellert BH, Bettany JR (1992). Temperature dependence of net nitrogen and sulfur mineralization. Soil Sci. Soc. Am. J..

[30] Myers RJK (1975). Temperature effects on ammonification and nitrification in a tropical soil. Soil Biol. Biochem..

[31] Sierra J (1997). Temperature and soil moisture dependence of N mineralization intact soil cores. Soil Biol. Biochem..

[32] Suszek-Łopatka B, Maliszewska-Kordybach B, Klimkowicz-Pawlas A, Smreczak B (2019). The drought and high wet soil condition impact on PAH (phenanthrene) toxicity towards nitrifying bacteria. J. Hazard. Mater..

[33] Suwa Y, Hattori T (1984). Effects of nutrient concentration on the growth of soil bacteria. Soil Sci. Plant Nutr..

[34] Jensen HL (1950). Effect of organic compounds on *Nitrosomonas*. Nature.

