# Supplementary material for: Development of soil-less substrates capable of degrading organic nitrogen into nitrate as in natural soils
Source: Sci Rep. 2022 Jan 17;12:785. doi: 10.1038/s41598-022-04873-0 (PMC8764028; doi:10.1038/s41598-022-04873-0)
Supplement: Supplementary file 1 — Supplementary Information. [file 41598_2022_4873_MOESM1_ESM.docx]

Development of soil-less substrates capable of degrading organic nitrogen into nitrate, as in natural soils

Jamjan Meeboon^1†^, Ryoya Nishida^1,2†^, Takashi Iwai^2^, Kazuki Fujiwara^1,3^, Masao Takano^2^, Makoto Shinohara^1^*

^1^ Institute of Vegetable and Floriculture Science, National Agriculture and Food Research Organization (NARO), 360 Ano, Tsu, Mie 514-2392, Japan

^2^Graduate School of Environmental Studies, Nagoya University, Furo-cho, Chikusa-ku, Nagoya, Aichi 464-8601, Japan

^3^Kyushu Okinawa Agricultural Research Center, NARO, 2421 Suya, Koshi, Kumamoto 861-1192, Japan

^†^Co–first authors

*Correspondence and requests for materials should be addressed to Makoto Shinohara (email: shsh@affrc.go.jp)

**Table S1. List of figures and tables.**

| Selection of carriers | Fig. 1 | Nitrogen mineralization by the microbial communities on different carriers. |
| --- | --- | --- |
|  | Table S2 | Physical properties of the porous carriers. |
|  | Fig. S1 | Plastic tubes packed with rockwool. |
|  | Fig. S2 | Fig. S2 Relationships between the efficiency of conversion of organic matter into nitrate-N and five physical properties of the carrier medium. WFPS, water-filled pore space. |
| Determination of optimal conditions | Fig. 2 | Comparisons between leachates from the inoculated rockwool carrier and leachates from the uninoculated rockwool. |
|  | Fig. 3 | Effect of excessive addition of organic substance and the resulting inorganic N composition. |
|  | Fig. 4 | Determination of optimal conditions for N mineralization in the leachate from rockwool treated with organic substances. Bars labelled with the same letter do not differ significantly (Fisher’s protected LSD test, *P* < 0.05). |
|  | Fig. S3 | Time course of inorganic N in leachate from the inoculated carrier over 30 days. |
|  | Fig. S4 | Effect of discontinuation of organic substance addition on the generation of inorganic N. |
|  | Fig. S5 | Time course of ammonium-N, nitrite-N, nitrate-N, phosphate, and potassium ions in leachate from the inoculated carrier during the first 32 days after inoculation. |
| Identification of microbes and confirmation of plant growth | Fig. 5 | Microbial phase analysis of the inoculated carriers, uninoculated material, and liquid “multiple parallel mineralization” (MPM) culture. |
|  | Fig. 6 | Plant growth on the inoculated carriers and the uninoculated materials. |
|  | Table S3 | Microbial-phase analysis. |
|  | Fig. S6 | Mineralization of organic N and its relationship with the microbial population density. |
|  | Fig. S7 | Plant growth in the inoculated carriers and in nursery soil. |

**Table S2. Physical properties of the porous carriers.**

Physical properties were measured as follows. First, 100 mL (bulk volume, *V*) of each material was prepared and weighed (*W*). The material was then transferred into a 500-mL graduated cylinder, which was filled to a depth of 100 mL with water. The amount of water added (*W*_W_) was measured. The water was then decanted, and the total amount discharged (*W*_f_) was measured. The dry weight (*W*_d_) of 100 mL of each material was measured after drying at 100 °C for 24 h. From the results, we calculated the specific gravity of each material, as well as its bulk density, solid phase ratio, liquid phase ratio after decantation, gas phase ratio after decantation, porosity, and water-filled pore space (WFPS), as follows:

Solid phase (%) = [{100 − *W*_W_ − (*W* − *W*_d_)}/*V*] × 100

Liquid phase (%) = [{*W* + (*W* − *W*_d_) − *W*_f_}/*V*] × 100

Gas phase (%) = 100 − (Solid phase) − (Liquid phase)

Specific gravity = (*W*_d_/100) / {(Solid phase)×*V*}

Bulk density (g/mL) = *W*_d_/100

Porosity (%) = (Liquid phase + Gas phase)

WFPS (%) = (Liquid phase) / (Porosity) × 100

**Table S3. Microbial-phase analysis.**

|  | Total sequences | *Arthrobacter* (%) | *Bacillus* (%) | *Nitrobacter* (%) | *Nitrospira* (%) | *Nitrosomonas* (%) |
| --- | --- | --- | --- | --- | --- | --- |
| Inoculated carrier | 32,130 | 38.1 | 0.05 | 0.3 | 0.01 | 0.2 |
| Bark compost | 38,114 | ND | 0.3 | 0.5 | 0.09 | 0.005 |
| MPM culture solution | 50,381 | 0.04 | 18.0 | 0.3 | 0.06 | 0.1 |

**
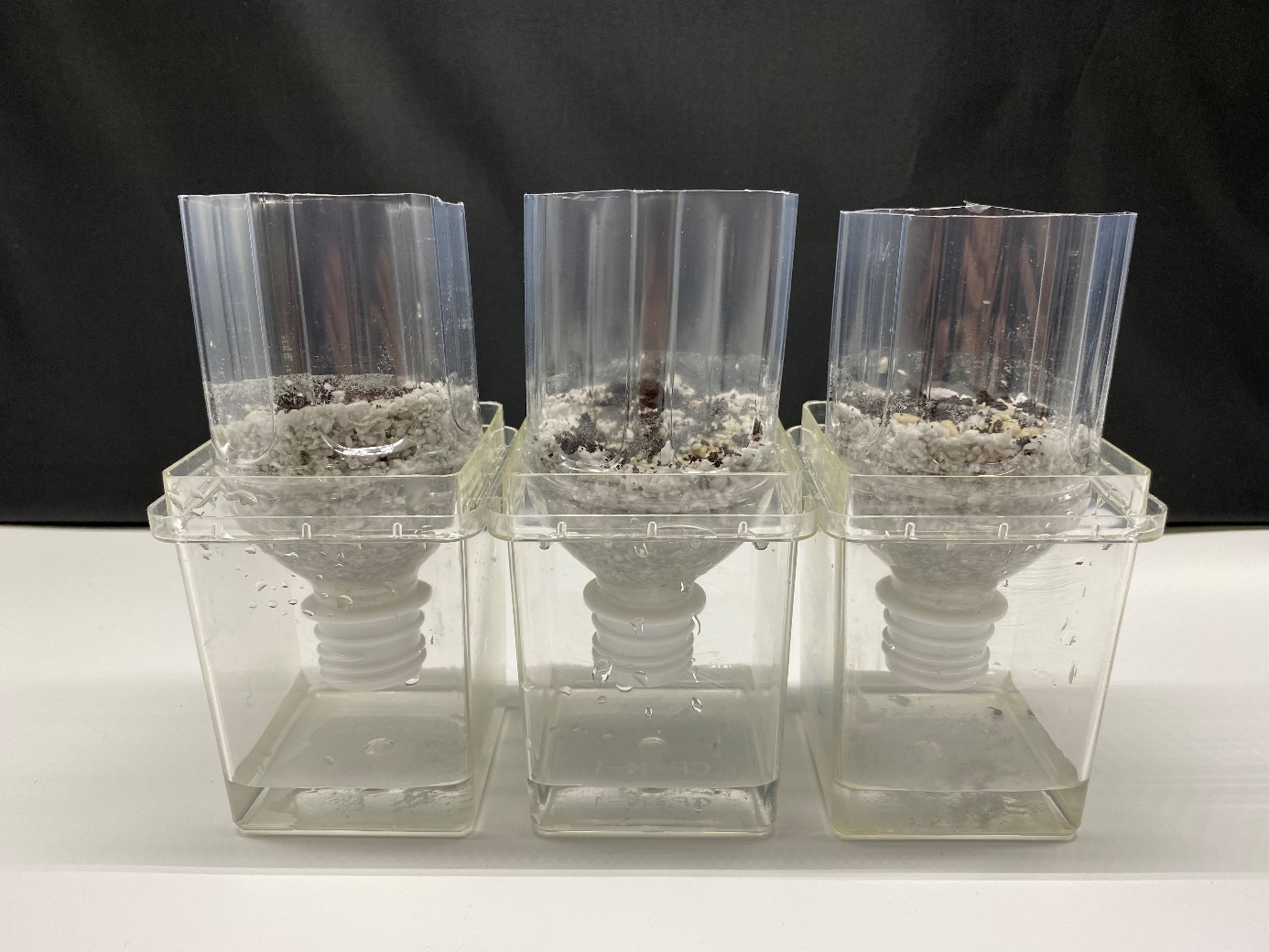
**

**Figure S1. Plastic tubes packed with rockwool.**


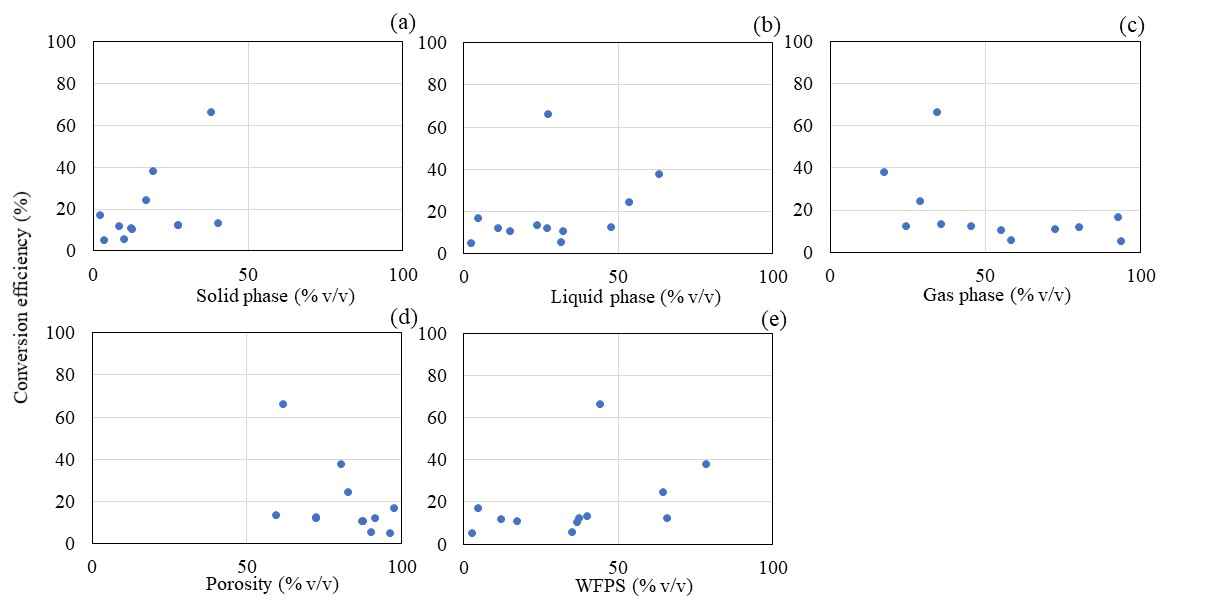


**Fig. S2 Relationships between the efficiency of conversion of organic matter into nitrate-N and five physical properties of the carrier medium. WFPS, water-filled pore space.**

The efficiencies of conversion to nitrate-N were based on the results in Fig. 1. Calculation of the physical properties was based on the data in Table S2. Pearson's correlation analysis revealed no significant correlation with efficiency of conversion into nitrate N for the solid phase ratio, liquid phase ratio, gas phase ratio, porosity, or WFPS (*P* > 0.05).

**
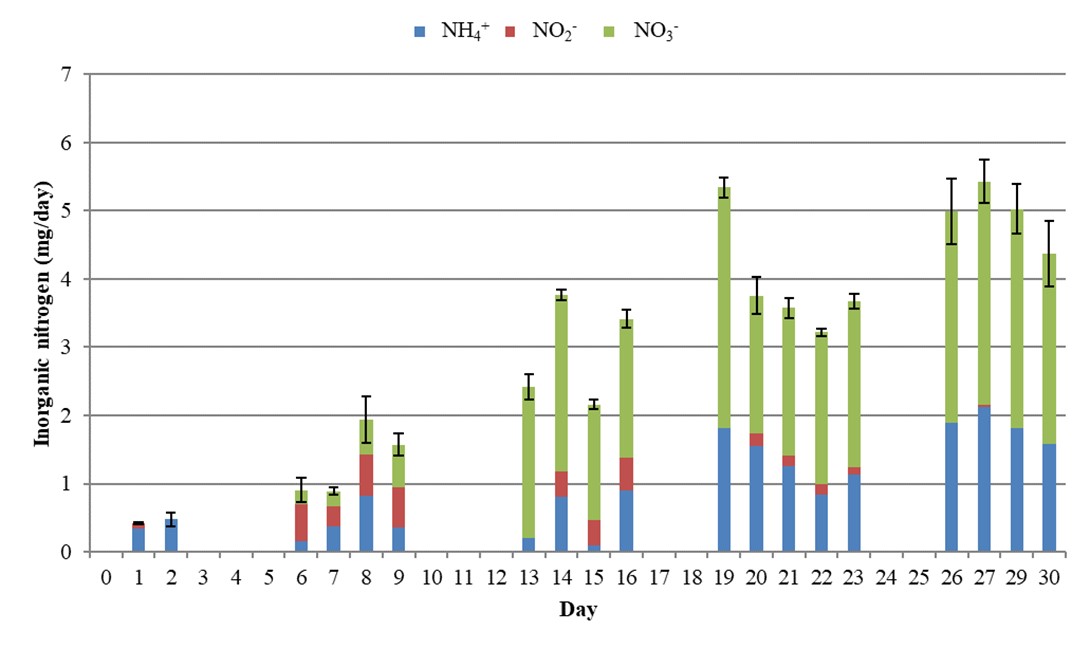
**

**Figure S3. Time course of inorganic N in leachate from the inoculated carrier over 30 days.**

Values are means ± SD (*n* = 3, Fisher’s protected LSD test, *P* < 0.05).


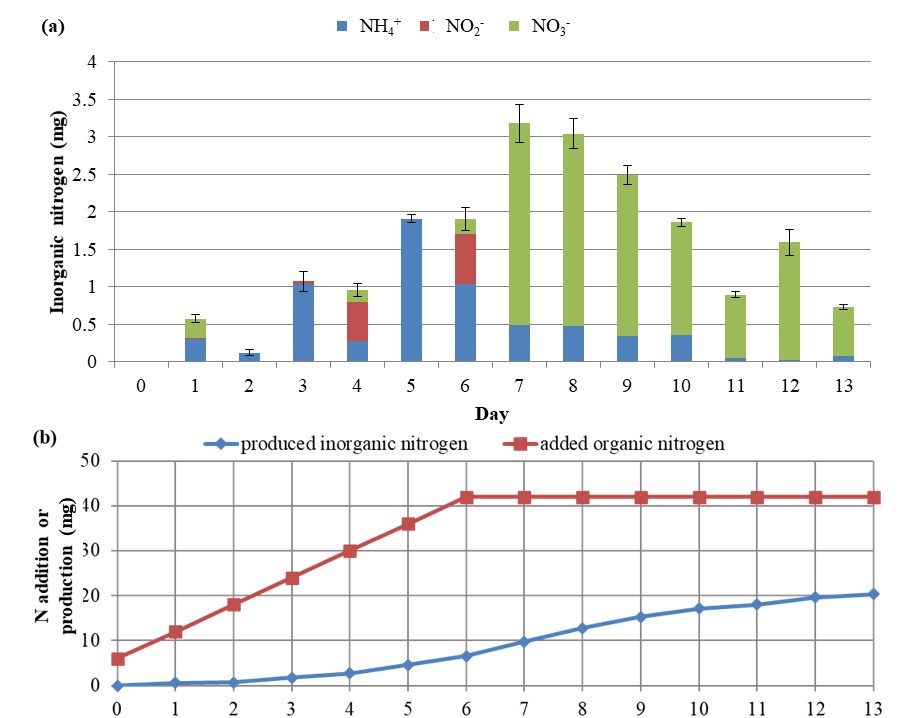


**Figure S4. Effect of discontinuation of organic substance addition on the generation of inorganic N.**

To 100 mL of rockwool, we added 1 g of bark compost as inoculant and 0.1 g of fish fertilizer as the organic substance. After incubation overnight at 25 °C, the rockwool was rinsed with 100 mL of water and then 0.1 g of fish fertilizer was added. The operation of adding fish fertilizer, incubating overnight at 25 °C, and rinsing with water was repeated 6 times, followed by water rinsing and incubation only. (a) Amount of inorganic N in each leachate solution. Values are means ± SD (*n* = 3, Fisher’s protected LSD test, *P* < 0.05). (b) Cumulative amounts of organic N added and of inorganic N recovered from the leachate. After the addition of the fish fertilizer, fertilizer was stopped on day 6, inorganic N continued to be produced at a decreasing rate, but the proportion of nitrate increased (a). This result shows a time lag between degradation of organic substances and the production of inorganic N.


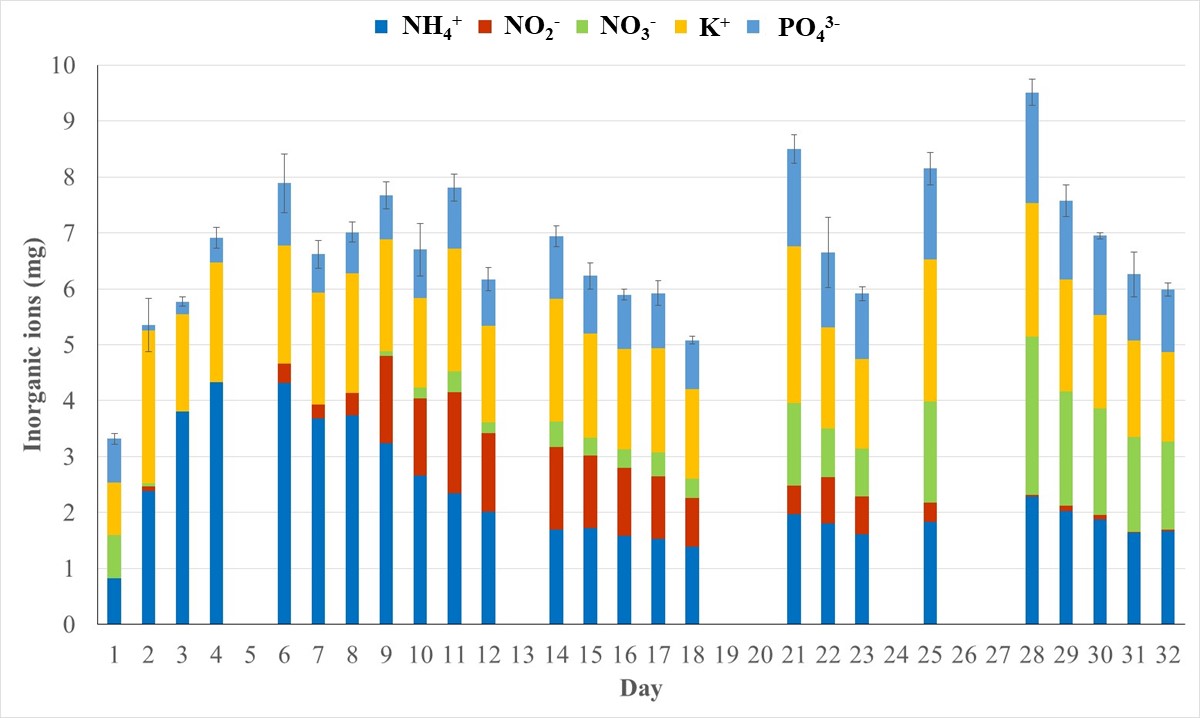


**Figure S5. Time course of ammonium-N, nitrite-N, nitrate-N, phosphate, and potassium ions in leachate from the inoculated carrier during the first 32 days after inoculation.**

Values are means ± SD (*n* = 3, Fisher’s protected LSD test, *P* < 0.05).


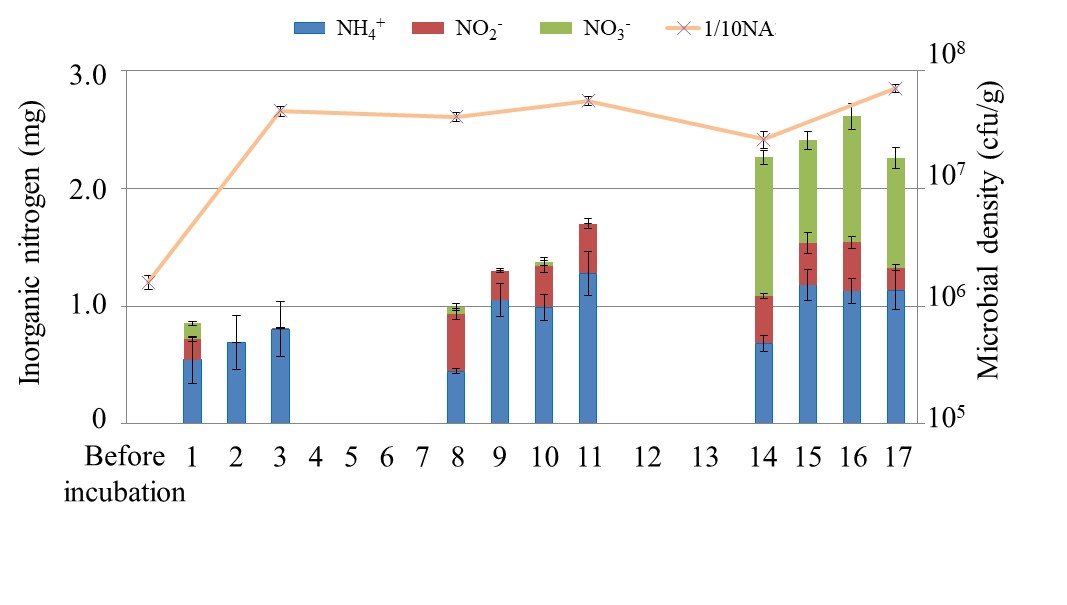


**Figure S6. Mineralization of organic N and its relationship with the microbial population density.**

Microbial densities in 1/10 NA medium and fish fertilizer and release of inorganic N from the inoculated carrier (rockwool). The population density increased dramatically from about 2 × 10^6^ cfu/g before incubation to about 60 × 10^6^ cfu/g after 3 days of incubation. During the first 3 days, mainly ammonium-N was produced. Subsequently, nitrite and nitrate began to appear, and the population increased to about 70 × 10^6^ cfu/g. These results show that the microbial population density increased with increasing nitrification. Values are means ± SD (*n* = 3, Fisher’s protected LSD test, *P* < 0.05).


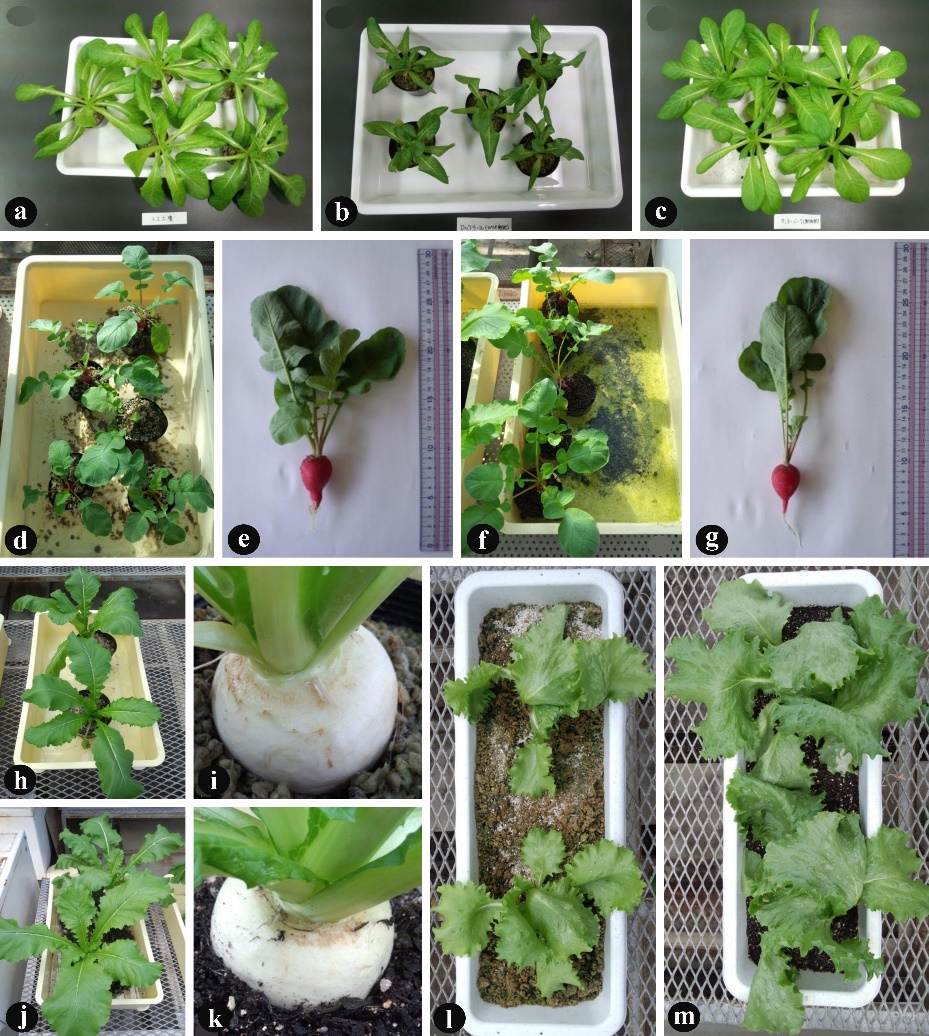


**Figure S7. Plant growth in the inoculated carriers and in nursery soil.**

Comparison of saladana lettuce growth in (a) inoculated carrier, (b) uninoculated material, and (c) soil, 26 days after sowing. (d–g) Radishes grown in (d, e) inoculated carrier and (f, g) soil, 36 days after sowing. (h–k) Turnips grown in (h, i) inoculated carrier and (j, k) soil, 43 days after sowing. (l, m) Ball lettuce grown in (l) inoculated carrier and (m) soil, 66 days after sowing.
